# Un syndrome malin des neuroleptiques compliqué d'hémorragie méningée et révélant une vascularite cérébrale

**DOI:** 10.11604/pamj.2014.19.247.5293

**Published:** 2014-11-05

**Authors:** Toufik Jouali, Brahim Boukatta, Brahim Bechri, Nawfel Houari, Abderahim Bouazzaoui, Hicham Sbai, Nabil Kanjaa

**Affiliations:** 1 Service de Réanimation Polyvalente A4, CHU Hassan II des Fès, Fès, Maroc

**Keywords:** Syndrome malin des neuroleptiques, vascularite cérébrale, rigidité, hyperthermie, neuroleptic malignant syndrome, cerebral vasculitis, rigidity;, hyperthermia

## Abstract

Le syndrome malin des neuroleptiques est une complication du traitement par les neuroleptiques. Son incidence est estimée à 0,02% dans la population générale. Le traitement reste symptomatique et repose essentiellement sur l'arrêt immédiat du traitement antérieur. Nous rapportons l'observation clinique d'une patiente de 26 ans, schizophrénique sous Chlorpromazine, se présentant aux urgences pour la prise en charge d'un syndrome malin des neuroleptiques compliqué d'une hémorragie méningée et révélant une vascularite cérébrale.

## Introduction

Décrit pour la première fois en 1960 par Delay et al sous le nom de syndrome akinétique et hypertonique [1] le syndrome malin des neuroleptiques (SMN) est une complication méconnue du traitement par les neuroleptiques. Son incidence dans la population générale est de 0,02% et sa mortalité varie de 10 à 20% [2]. C'est une urgence diagnostic et impose un transfert en une unité de soins intensifs. L'objectif de ce travail est de rapporter un cas d'hémorragie méningée compliquant un syndrome malin des neuroleptiques chez une patiente porteuse d'une vascularite cérébrale méconnue.

## Patient et observation

**Figure 1:**
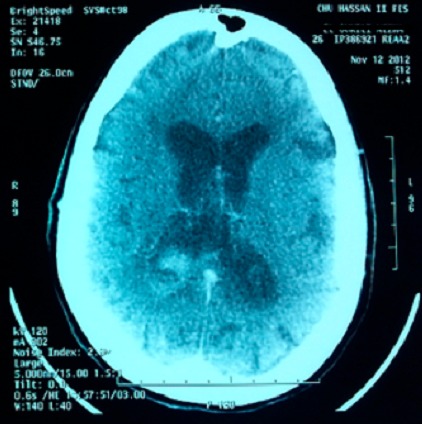
Coupe scannographique montrant une hémorragie méningée avec hémorragie intra-ventricule

**Figure 2:**
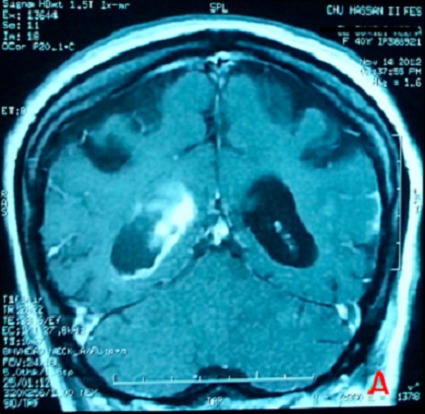
Coupe coronale en IRM objectivant une hémorragie intra-ventriculaire en T1

**Figure 3:**
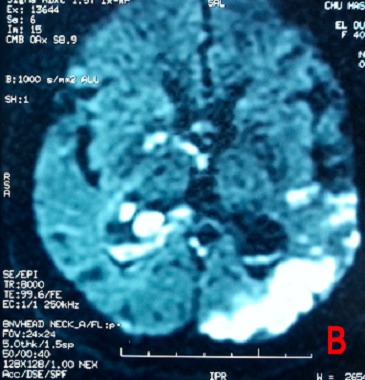
Coupe transversale en IRM au Flair objectivant des lésions d'ischémie cortico-sous corticale

**Figure 4:**
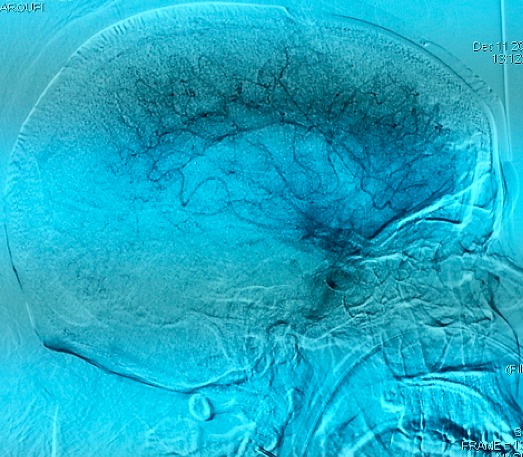
Image d'artériographie cérébrale faisant évoquer des lésions de vascularite cérébral

Mme O.N, patiente de 26 ans, suivie en psychiatrie pour une schizophrénie sous traitement depuis un mois par Chlorpromazine, est admise aux urgences dans un tableau de trouble de conscience fébrile. L'examen clinique a trouvé une patiente avec un Glasgow à 11, une hyperthermie à 39,5 avec une rigidité généralisée, une hypersudation, un encombrement bronchique et une dyspnée inspiratoire faisant évoquer en premier lieu un syndrome malin des neuroleptiques. La patiente est admise en réanimation avec l'arrêt immédiat des neuroleptiques et la prise en charge consiste à une hyper-réhydratation, une administration de Diazépam pour la rigidité, une oxygénothérapie, les moyens antipyrétiques (paracétamol, vessies de glace...) et une prévention thrombo-embolique après un scanner cérébral normal. Un traitement par la Nicardipine à la seringue auto-pulsée est également instauré pour la gestion des pics hypertensifs. A J+3 de son hospitalisation, la patiente aggrave son Glasgow devenant à 8 indiquant son intubation. Un scanner cérébral réalisé objective une hémorragie méningée avec hémorragie intra-ventriculaire (Figure 1). Le bilan étiologique est complété par une IRM cérébrale objectivant des lésions d'ischémie cortico sous corticale à droite avec un hématome intra-ventriculaire faisant évoquer des lésions de vascularite cérébrale (Figure 2, Figure 3) confirmée par la suite par une artériographie cérébrale (Figure 4).

Sur le plan biologique on note une rhabdomyolyse à j+2 de son hospitalisation avec des CPK à 4199mg/l, une légère insuffisance rénale fonctionnelle avec une urée 0,62 g/l à et une créatinémie à 11mg/l, une cytolyse hépatique à trois fois la normale, des leucocytes à 22000 elt/ml, une CRP à 72mg/l et le reste du bilan est normal notamment la ponction lombaire revenant en faveur d'une hémorragie méningée. Le traitement était symptomatique avec la perfusion de la Nimodipine pour l'hémorragie méningée. L’évolution était favorable sur 43 jours d'hospitalisation marquée par une trachéotomie pour sevrage respiratoire à J+10 et par la survenue d'une pneumopathie nosocomiale à *Acinétobacter Baumani*. La patiente est transférée par la suite en neurologie pour diagnostic de sa vascularite et le complément de prise en charge après sa récupération complète du SMN, la normalisation de son bilan biologique et le traitement de sa pneumopathie nosocomiale.

## Discussion

Evoqué pour la première fois en 1960 par Delay et Denker, le syndrome malin des neuroleptiques est un effet indésirable rare mais potentiellement mortel lié à l'utilisation des antipsychotiques et des médicaments altérant la neurotransmission dopaminergique [1]. Son incidence est estimée actuellement à 0,02% parmi les patients sous traitement neuroleptique [2]. Les hommes sont deux fois plus touchés que les femmes, les jeunes plus que les vieux [3] et actuellement la mortalité est de 10 à 20% par rapport à 75% de mortalité décrite dans les premiers cas évoqués dans la littérature [4]. Selon nos connaissances, jusqu’à présent aucun cas d'association du SMN et d'hémorragie méningée n'a été rapporté dans la littérature. La survenue du syndrome est idiosyncrasique [5]. Toutefois, les symptômes apparaissent dans deux tiers des cas dans les deux premières semaines suivant l'introduction d'un neuroleptique ou l'augmentation de la dose d'un traitement en cours [6, 7]. Chez notre malade, le SMN s'est manifesté un mois après le début du traitement par la Chlorpromazine et le tableau clinique était typique et fait de rigidité musculaire importante, de fièvre en plateau malgré les moyens physiques avec une altération de l’état de conscience et une tachycardie et pics hypertensifs [3]. La survenue de l'hémorragie méningée chez notre malade pourrait être une complication des pics hypertensifs de la dysautonomie du SMN et elle serait favorisée par la présence de la vascularite cérébrale méconnue. D'un autre côté, les vascularites cérébrales peuvent être associées et/ou se manifester par de multiples troubles psychiatriques, notamment dans le cas des connectivités et des maladies de système [8], d'où la remise en question du diagnostic de la schizophrénie chez notre malade qui pourrait être une manifestation de la vascularite qui reste méconnue avec un bilan immunologique non concluant.

La physiopathologie du SMN repose sur deux théories. La première est périphérique est c'est l'effet de la libération du calcium dans le réticulum sarcoplasmique des cellules musculaires striées provoquant ainsi une contraction musculaire permanente et une augmentation de la température corporelle [9]. La deuxième théorie est centrale et c'est l'effet de l'activité anti-dopaminérgique centrale des neuroleptiques stimulant le système cholinergique et induisant un syndrome extrapyramidal (rigidité, tremblement) [10]. Le diagnostic différentiel du SMN est fait essentiellement avec le syndrome sérotoninergique et avec les causes infectieuses et toxiques d'atteinte nerveuse centrale [11]. L’évolution est intimement liée à la rapidité d’établissement du diagnostic et la suspicion d'un syndrome malin impose, en premier lieu, l'arrêt immédiat du traitement neuroleptique [12]. Dans notre cas, après l'arrêt des neuroleptiques, la prise en charge a comporté une rééquilibration hydro-électrolytique, une lutte active contre l'hyperthermie par vessies de glace et draps réfrigérés, une nutrition correcte et une prévention thromboembolique. La nicardipine a été utilisée initilament pour la gestion des pics hypertensifs puis reliée par la perfusion de la nimodipine pour l'hémorragie méningée. Une assistance ventilatoire était nécessaire avec une trachéotomie à j+10 pour le sevrage respiratoire avec le traitement de la pneumopathie nosocomiale. Les traitements pharmacologiques spécifiques, type Bromocriptine et Dantrolène, n'ont pas été instaurés et la récupération neurologique était plus allongée que ce qui est décrit dans la littérature du fait de la survenue de l'hémorragie méningée. La reprise du traitement neuroleptique doit être discutée attentivement car les patients ayant un antécédent de syndrome malin, présentent plus de risque d'en refaire un autre avec un taux de récurrence de 30% [1, 2, 7]. Dans notre cas, la reprise du traitement s'est faite au bout de quatre semaines, à moitié dose avec une surveillance rapprochée de la patiente et sans incident particulier.

## Conclusion

Avec l'hémorragie méningée comme complication de plus, aussi rare qu'il puisse l’être, le syndrome malin des neuroleptiques est une complication du traitement par les neuroleptiques dont l’évolution peut être grave avec une morbi-mortalité assez importante. Le faible nombre de cas rapportés et l'absence de consensus actuel autour des critères diagnostiques, rendent les études contrôlées et la compréhension physiopathologique de cette entité difficiles.
